# Leprosy in China

**Published:** 1846-12

**Authors:** 


*Leprosy in China.*—It is reported that leprosy is exceedingly common among the Chinese, and that it is very prevalent at Canton. Leper hospitals now exist there as they did in England up to the end of the 15th century. It is said, however, that they are over-crowded with patients; and, as there is a great horror of the disease, many are turned out and allowed to die on the highways and in the ditches. The entire absence of cleanliness among the lower class of Chinese will sufficiently account for the prevalence of this and other diseases of the skin among them.—*Lond. Med. Gaz.*

